# The centrally restricted diffusion sign on MRI for assessment of radiation necrosis in metastases treated with stereotactic radiosurgery

**DOI:** 10.1007/s11060-021-03879-4

**Published:** 2021-10-24

**Authors:** Nicolin Hainc, Noor Alsafwani, Andrew Gao, Philip J. O’Halloran, Paul Kongkham, Gelareh Zadeh, Enrique Gutierrez, David Shultz, Timo Krings, Paula Alcaide-Leon

**Affiliations:** 1 Department of Medical Imaging, University of Toronto, Toronto, Canada; 2 Department of Neuroradiology, Clinical Neuroscience Center, University Hospital Zurich, University of Zurich, Frauenklinikstrasse 10, 8091 Zurich, Switzerland; 3 Laboratory Medicine Program, University Health Network, Toronto, Canada; 4 Department of Pathology, College of Medicine, Imam Abdulrahman Bin Faisal University (IAU), Dammam, Saudi Arabia; 5 Neurosurgery, University Health Network, Toronto, Canada; 6 Radiation Oncology, Princess Margaret Cancer Centre, Toronto, Canada; 7 Joint Department of Medical Imaging, University Health Network, Toronto, Canada

**Keywords:** Diffusion magnetic resonance imaging, Radiation surgery, Radiation injuries, Necrosis

## Abstract

**Purpose:**

Differentiation of radiation necrosis from tumor progression in brain metastases treated with stereotactic radiosurgery (SRS) is challenging. For this, we assessed the performance of the centrally restricted diffusion sign.

**Methods:**

Patients with brain metastases treated with SRS who underwent a subsequent intervention (biopsy/resection) for a ring-enhancing lesion on preoperative MRI between 2000 and 2020 were included. Excluded were lesions containing increased susceptibility limiting assessment of DWI. Two neuroradiologists classified the location of the diffusion restriction with respect to the post-contrast T1 images as centrally within the ring-enhancement (the centrally restricted diffusion sign), peripherally correlating to the rim of contrast enhancement, both locations, or none. Measures of diagnostic accuracy and 95% CI were calculated for the centrally restricted diffusion sign. Cohen's kappa was calculated to identify the interobserver agreement.

**Results:**

Fifty-nine patients (36 female; mean age 59, range 40 to 80) were included, 36 with tumor progression and 23 with radiation necrosis based on histopathology. Primary tumors included 34 lung, 12 breast, 5 melanoma, 3 colorectal, 2 esophagus, 1 head and neck, 1 endometrium, and 1 thyroid. The centrally restricted diffusion sign was seen in 19/23 radiation necrosis cases (sensitivity 83% (95% CI 63 to 93%), specificity 64% (95% CI 48 to 78%), PPV 59% (95% CI 42 to 74%), NPV 85% (95% CI 68 to 94%)) and 13/36 tumor progression cases (difference p < 0.001). Interobserver agreement was substantial, at 0.61 (95% CI 0.45 to 70.8).

**Conclusion:**

We found a low probability of radiation necrosis in the absence of the centrally restricted diffusion sign.

## Introduction

Brain metastases are the most common form of intracranial tumor in the adult population and their incidence appears to be increasing due to longer patient survival and improving imaging techniques [1, 2]. Stereotactic radiosurgery (SRS) is a treatment mainstay in patients with limited (< 5) metastases and is increasingly being used in patients with multiple (≥ 5) metastases [3–5]. Assessing treatment response after SRS remains a challenge as a lesion size increase can be seen in up to 32% of radiated metastases, representing either radiation necrosis or true progression [6], and differentiation of these two entities is difficult based on conventional imaging alone [7].

Radiation necrosis can be characterized by a transient or progressive increase in enhancing lesion size due to inflammatory changes, and mimics tumor progression on imaging [8, 9]. These lesions may stabilize or subside without additional therapy but imaging diagnosis usually requires several months of follow-up [10, 11]. In the case of progression, further treatment including surgical resection should be considered early for optimal local control [12]. While surgery is indicated in some cases of radiation necrosis, other cases in which it is mistaken for progression will result in unnecessary procedures or further radiation, which carry their own inherent risks, or suboptimal systemic therapies tailored to true progression [13]. Multiple advanced imaging techniques including dynamic contrast enhanced (DCE) and dynamic susceptibility contrast (DSC) MR-perfusion, MR-spectroscopy, intravoxel incoherent motion perfusion (IVIM) [14–22], and nuclear medicine studies including 18F-fluoro-ethyl-l-tyrosine positron emission tomography (18F-FET PET) [23] and 11C-methionine PET (11C MET-PET) [24] have been applied to differentiate radiation necrosis from tumor progression.

Diffusion weighted imaging (DWI) and the derived apparent diffusion coefficient (ADC) is a biomarker measuring water mobility in tissue providing indirect information on the tissue micro-environment [25–27]. Previous studies on brain metastases after SRS using ADC are based on quantitative differences on serial follow up imaging or require time-consuming lesion component segmentation [14, 25, 28, 29]. In a recent study on high-grade gliomas, Zakhari et al. [30] describe central diffusion restriction within a ring enhancing lesion to be indicative of radiation necrosis on visual analysis alone, with a study by Alcaide et al. validating this finding [31].

The aim of this study was to assess the diagnostic accuracy of the centrally restricted diffusion sign to differentiate radiation necrosis from tumor progression in patients with brain metastases treated with SRS, with ground truth determined by histopathology.

## Materials and methods

### Subjects

Institutional Review Board approval was obtained for this single center retrospective study. A Laboratory Information System search and Neurosurgery Archive search was performed using keywords “radiation”, “treatment”, and “metastasis” from 2000–2020. We included patients with SRS treated brain metastasis demonstrating a ring-enhancing lesion with central necrosis (on MRI) who subsequently underwent resection or biopsy. Excluded were patients without available pre-surgical/biopsy MRI studies in our picture archiving and communication system (PACS). Furthermore, patients lacking central necrosis on MRI were excluded. Central necrosis on MRI was defined as a nonenhancing region surrounded by contrast enhancement with the sum of biperpendicular diameters > 10 mm. Finally, patients with increased susceptibility within the lesions were also excluded as assessment of DWI is limited in these cases [32].

### MR acquisition

MR examinations were performed on 1.5 T and 3 T scanners (General Electric Healthcare, Waukesha, WI and Siemens Healthineers AG, Erlangen, Germany) using an eight-channel phased-array head coil. T1 weighted sequences were acquired before and after a bolus injection of 0.1 mmol/kg body weight Gadobutrol (Gadovist, BayerHealthCare, Berlin, Germany). For T1 on GE, the volumetric T1-weighted fast spoiled grass sequence with IR preparation (IR-FSPGR) was acquired with following parameters: TR/TE = 7.22–9.00/1.48–4.20 ms, matrix = 256 × 224–256, slice thickness = 1.0–2.0 mm, FOV = 24–25 × 24–25 cm. For T1 on Siemens, the volumetric T1-weighted magnetization prepared rapid gradient echo (MP-RAGE) was acquired with following parameters: TR/TE = 2200.00/2.47 ms, matrix = 256 × 256, slice thickness = 1.0 mm, FOV 25 × 25 cm. DWI sequence parameters: TR/TE = 5600.00–9000.00/69.20–94.00 ms, matrix = 128–256 × 128–232, slice thickness = 4.0–5.0 mm, FOV = 19.9–26 cm × 22–26 cm, b = 1000 s/mm^2^. ADC maps were generated from diffusion images. For GE, the T2*-weighted sequence was acquired with following parameters: TR/TE = 4000.00/30.00 ms, FOV = 22–24 × 22–24, matrix = 384 × 256, slice thickness = 5 mm. For Siemens, the susceptibility weighted image (SWI) sequence was acquired with following parameters: TR/TE = 49.00/40.00, matrix = 256 × 177, slice thickness = 2.0–2.6 cm, FOV = 20.1 × 23.0 cm.

### Image interpretation

Screening of patients for inclusion in the study was performed by two neuroradiologists with 10 years and 7 years of experience (PA-L and NH). Patients demonstrating susceptibility artifact due to blood products within the treated lesion or lacking an area of necrosis were excluded. For the included patients, readers individually evaluated all cases for presence and location of the diffusion restriction using b1000 trace images and ADC maps in conjunction with post contrast T1 images in order to classify the lesions according to four different patterns. Disagreements were resolved by consensus. A hyperintensity on b1000 trace images with corresponding low ADC, lower than normal appearing white matter, was considered restricted diffusion. Diffusion restriction found within the boundaries of the ring enhancing lesion defined the centrally restricted diffusion sign. Four different diffusion patterns were thus possible and were categorized as follows: (1) “central” i.e. centrally within the ring enhancing lesion (the centrally restricted diffusion sign), (2) “peripheral” i.e. peripherally correlating only to the rim of contrast enhancement, (3) “both” i.e. diffusion restriction both centrally and peripherally (which thus includes central diffusion restriction and is counted as containing the centrally restricted diffusion sign), and (4) “no” i.e. no diffusion restriction.

True positives, true negatives etc. are defined based on the presence or absence of the centrally restricted diffusion sign on MRI with histopathology taken as the ground truth. Cases of histopathological radiation necrosis containing the centrally restricted diffusion sign on MRI, either through pattern 1 “central” or pattern 3 “both,” are counted as true positive cases. Cases of histopathological tumor progression containing these patterns are counted as false positive cases. Conversely, cases of histopathological tumor progression not containing the centrally restricted diffusion sign on MRI, either through pattern 2 “peripheral” or pattern 4 “no” are counted as true negative cases. Cases of histopathological radiation necrosis containing these patterns are counted as false negative cases.

### Histopathology interpretation

All specimens were fixed in formalin with routine preparation of hematoxylin and eosin-stained slides. Final diagnosis of radiation necrosis or tumor progression was made by expert pathologists based on the presence or absence of viable tumor with possible perivascular or intravascular distributions, necrotic tumor, fibrinoid necrosis, hemorrhage, hyalinization and thrombosis of the blood vessels, foamy macrophages, hemosiderin-laden macrophages, reactive gliosis, dystrophic calcification, and ghost cells, as was described in previous studies focusing on SRS of brain metastases [33, 34]. For subanalysis of all tumor progression cases, percentages of tumor cells for cases with and without the centrally restricted diffusion sign were recorded.

### Statistical analysis

Interobserver agreement among the four different diffusion patterns was identified using Cohen's kappa [35]. A κ value of 0.2 indicates slight agreement; 0.21–0.4 fair agreement; 0.41–0.6 moderate agreement; 0.61–0.80 substantial agreement, and 0.81–1.00 almost perfect agreement [36]. Measures of diagnostic accuracy and 95% CI were calculated for the centrally restricted diffusion sign indicating radiation necrosis. To investigate the performance of the centrally restricted diffusion sign to differentiate radiation necrosis from tumor progression, the two-sided Fisher’s exact test was performed. To further assess the tumor progression group by comparing cases containing (i.e. false positives) and not containing (i.e. true negatives) the centrally restricted diffusion sign, the Mann–Whitney *U* test was performed for variables including histopathologic tumor fraction, patient age, days from SRS to MRI, radiation dose, and prior WBRT through comparison of centrally restricted and non-centrally restricted tumor progression cases. Significance was set to p < 0.05. Statistical analyses were performed using GraphPad Prism 8.2.1.

## Results

### Patients

**Fig. 1:**
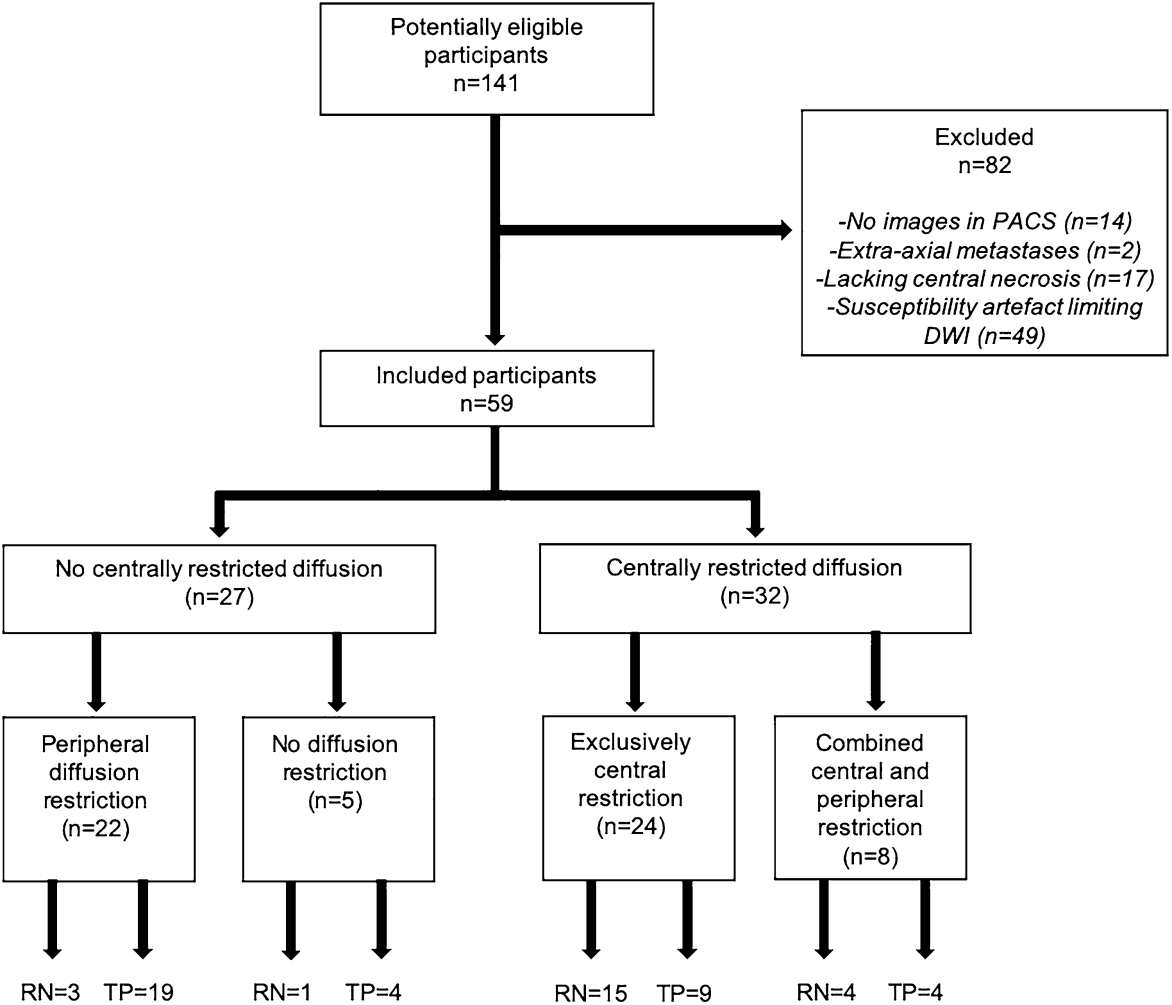
Standards for the reporting of diagnostic accuracy studies flow diagram. *RN* radiation necrosis, *TP* tumor progression

One hundred forty one patients with brain metastases treated with SRS who underwent a subsequent intervention (biopsy/resection) between February 2000 to February 2020 were retrospectively reviewed. After excluding patients without preoperative MRI in our PACS (n = 14), with extra-axial metastases (n = 2), lacking areas of necrosis (n = 17) or demonstrating susceptibility artefact limiting DWI assessment (n = 49), a total of 59 patients were included (36 female and 23 male) with a mean age of 59 years (range 40 to 80). Primary tumor histology was determined as follows: 34 lung, 12 breast, 5 melanoma, 3 colorectal, 2 esophagus, 1 head and neck, 1 endometrium, and 1 thyroid. Surgical resection was performed in 58 patients, while biopsy was performed in one patient. Mean time period from SRS to imaging was 403 days (SD 226, range 71 to 1001 days). Mean time period from SRS to surgery was 410 days (SD 223, range 71 to 1001 days). The date of SRS was available for 52/59 patients. Mean SRS dose was 18 Gy (SD 3, range 10 to 27 Gy). Dose information was available for 44/59 patients. 18 patients had additional whole brain radiotherapy (WBRT) prior to SRS, 2 concomitant, and 5 following SRS. One patient had prior SRS to the same site. Thirteen patients underwent cavity SRS following initial surgical resection (4 with progressive tumor, 9 with radiation necrosis). The Standards for the Reporting of Diagnostic Accuracy Studies (STARD) flow diagram is shown in Fig. 1.

### Predictive value of the patterns

**Fig. 2:**
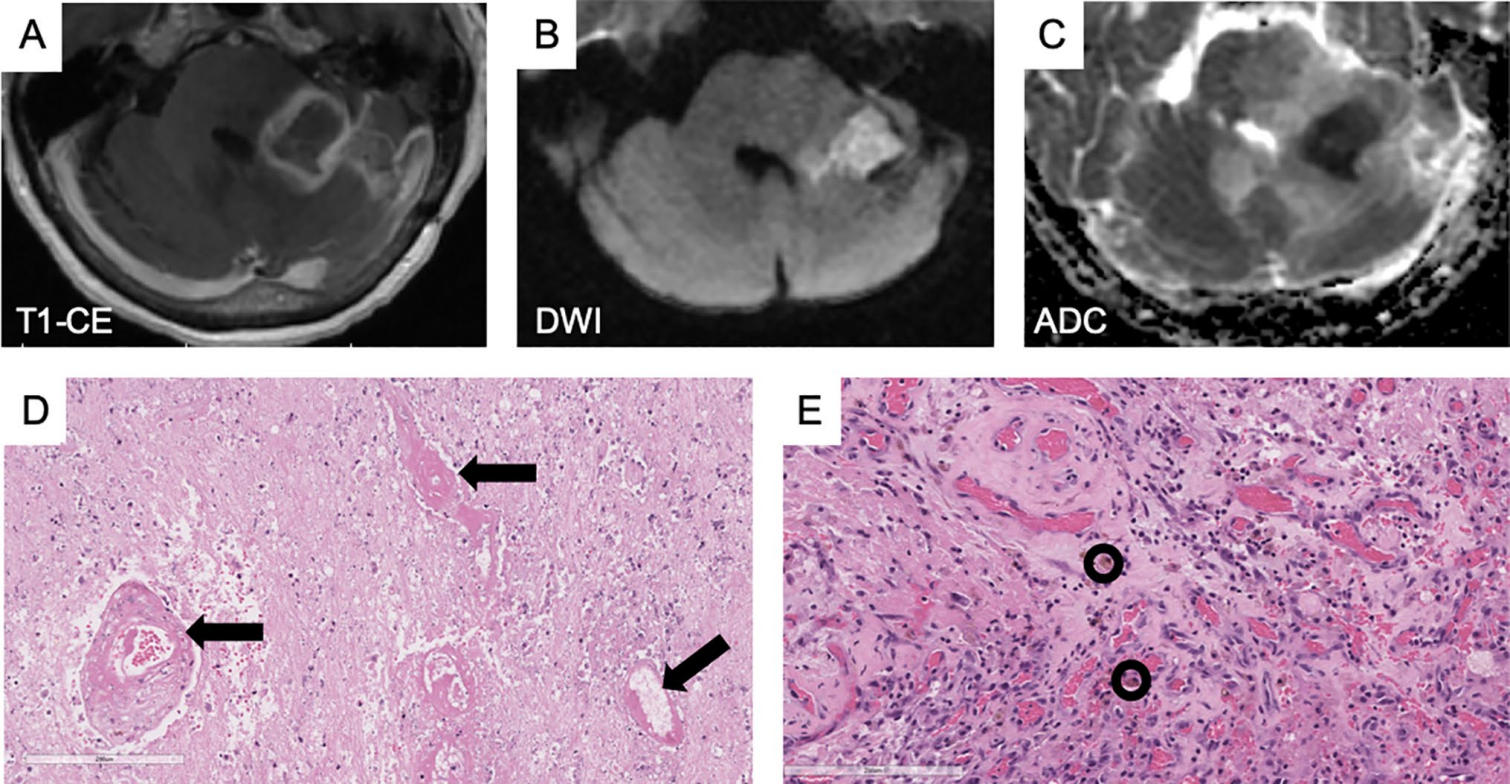
Histopathologic correlation of a surgically resected lesion containing the centrally restricted diffusion sign, involving the left middle cerebellar peduncle, determined to represent radiation necrosis. **A** Contrast enhanced T1-weighted image demonstrating ring enhancement. **B** Diffusion weighted image and **C** ADC map demonstrating restricted diffusion within the ring enhancing lesion. Corresponding hematoxylin and eosin stained sections showing morphologic features of radiation effects composed of **D** coagulative-type necrosis and vasculopathic changes including angionecrosis, mural hyalinization, and luminal stenosis (black arrows). **E** There are aggregates of vascular proliferation with hemosiderin-laden macrophages (black circles)

**Fig. 3:**
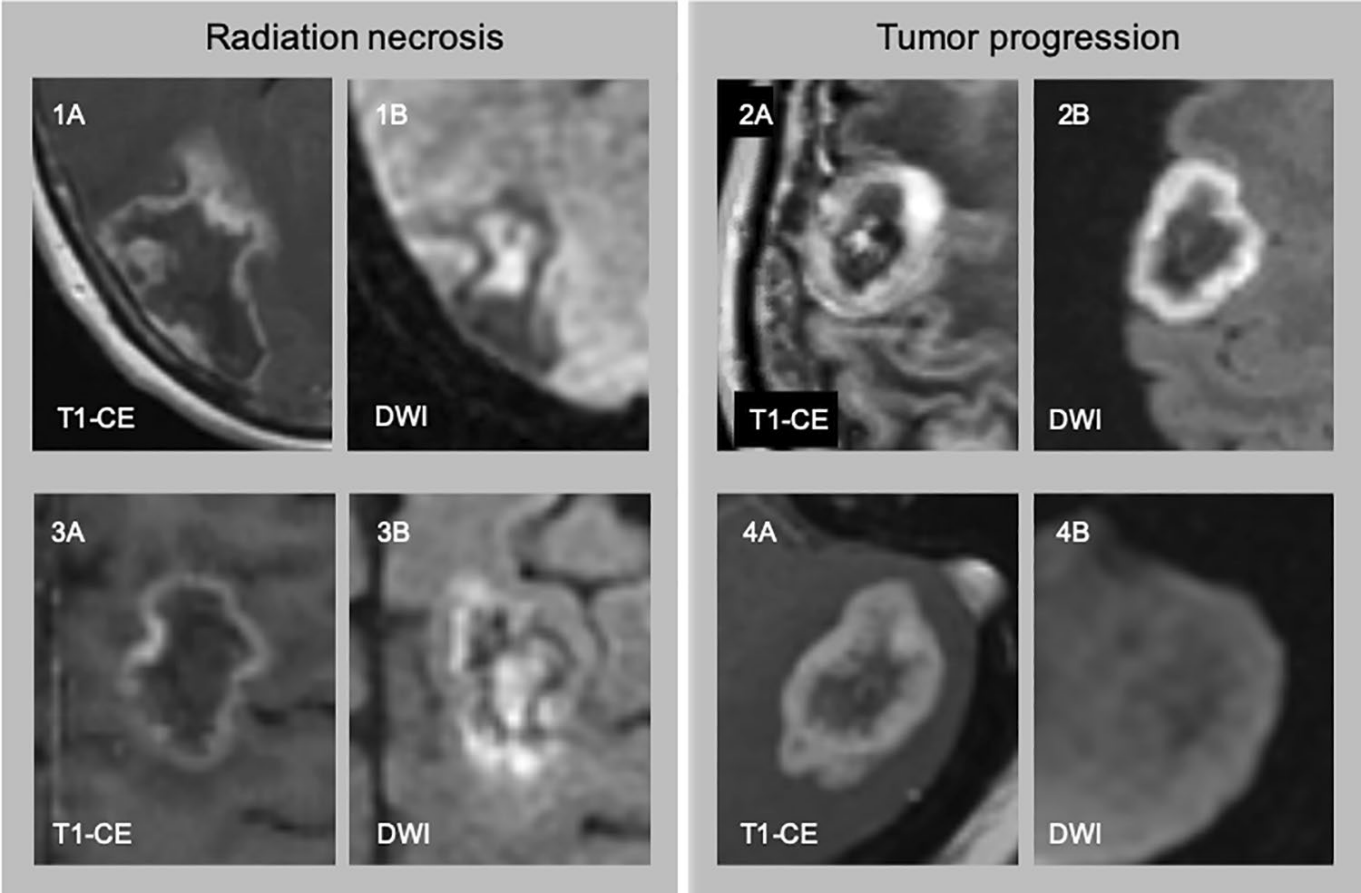
The four diffusion patterns. 1A: a ring enhancing lesion (T1-CE) with central diffusion restriction (1B, DWI), “central” pattern, histologic radiation necrosis; 2A: a ring enhancing lesion (T1-CE) with peripheral diffusion restriction (2B, DWI) overlapping the enhancing component, “peripheral” pattern, histologic tumor progression; 3A a ring enhancing lesion (T1-CE) with central and peripheral diffusion restriction (3B, DWI), “both” pattern, histologic radiation necrosis; 4A: a ring enhancing lesion (T1-CE) without associated diffusion restriction (4B, DWI), “no” pattern, histologic tumor progression

Of the 59 included patients, 23 (39%) were determined to have radiation necrosis by histopathology with the remaining 36 (61%) determined to have tumor progression. Of the radiation necrosis cases, the centrally restricted diffusion sign was found in 19/23 (83%) cases (Fig. 2). This included 15 cases with central diffusion restriction, i.e. exclusively within the ring enhancing lesion, and 4 with central diffusion restriction combined with peripheral diffusion restriction (i.e. overlapping the ring enhancement) (Table 1). The four cases not demonstrating either of these two patterns included one with no diffusion restriction and three with peripheral diffusion restriction only. Examples of the four diffusion patterns described are shown in Fig. 3.

**Table 1:** Number of subjects with radiation necrosis and tumor progression based on histopathology and the corresponding findings on MRI

Histopathology qround truth)	Central restriction (MRI)	No central restriction (MRI)
Radiation necrosis n = 23	19 True positive cases 15 “central” pattern 4 “both” pattern	4 False negative cases 3 “peripheral” pattern 1 “no” pattern
Tumor progression n = 36	13 False positive cases 9 “central” pattern 4 “both” pattern	23 True negative cases 19 “peripheral” pattern 4 “no” pattern

True positives, false positives, true negatives, and false negatives are defined within the table with histopathology taken as the ground truth

For tumor progression cases, peripheral diffusion restriction was seen in 19/36 (53%) cases and no diffusion restriction was seen in 4/36 (11%) cases. The remaining 13 (36%) cases were false positives containing the centrally restricted diffusion pattern (9 central, 4 central and peripheral).

The centrally restricted diffusion sign had a sensitivity of 83% (95% CI 63 to 93%) and a specificity of 64% (95% CI 48 to 78%) for radiation necrosis, with a positive predictive value (PPV) of 59% (95% CI 42 to 74%), and a negative predictive value (NPV) of 85% (95% CI 68 to 94%). The presence of the centrally restricted diffusion sign significantly differentiated radiation necrosis from tumor progression (p < 0.001).

### Interobserver agreement

The κ value was 0.61 (95% CI 0.45 to 70.8) for the differentiation of the four diffusion patterns.

### Subanalysis of tumor progression cases

The relatively high number of tumor progression cases with central diffusion restriction (13/36 (36%), i.e. false positives) prompted further subanalysis. In a subgroup analysis differentiating among tumor progression cases with the centrally restricted diffusion sign (i.e. false positives; 7 lung, 4 breast, 1 melanoma, 1 head and neck) and without the centrally restricted diffusion sign (i.e. true negatives; 13 lung, 4 breast, 2 colorectum, 2 esophagus, 1 melanoma, 1 endometrium) there was no difference in histopathologic tumor fraction (38 ± 21% vs 46 ± 23%), patient age (62 ± 9 years vs 60 ± 11 years), days from SRS to MRI (392 ± 221 days vs 415 ± 236 days), radiation dose (17.8 ± 5.0 Gy vs 17.8 ± 2.7 Gy), or prior WBRT (38% of cases vs 52% of cases) (Table 2).

**Table 2:** Assessment of all Tumor Progression cases comparing false positives (central diffusion restriction) and true negatives (no central diffusion restriction)

Tumor progression cases n = 36	Central restriction n = 13 (false positive cases)	No central restriction n = 23 (true negative cases)	p value
Histopathologic tumor Fraction (%)	38 ± 21	46 ± 23	> 0.05
Patient age (years)	62 ± 9	60 ± 11	> 0.05
Time from SRS to MRI (days)	392 ± 221	415 ± 236	> 0.05
SRS dose (Gy)	17.8 ± 5.0	17.8 ± 2.7	> 0.05
Additional WBRT (n)	5/13 (38%)	12/23 (52%)	> 0.05
Primary tumor (n)	Lung (7) Breast (4) Melanoma (1) Head and neck (1)	Lung (13) Breast (4) Colorectum (2) Esophagus (2) Melanoma (1) Endometrium (1)	

*WBRT* whole brain radiotherapy

No significant differences were found between the two groups

## Discussion

Our study demonstrates a low probability of radiation necrosis in SRS treated brain metastases in the absence of the central diffusion restriction sign, with a negative predictive value of 85.2%, a sensitivity of 83%, and a specificity of 64%. This is comparable to advanced MR imaging techniques for differentiating radiation necrosis from tumor progression including DSC Perfusion (rCBV) (Hoefnagels et al. sensitivity 70%, specificity 92.9% [15], Barajas et al. sensitivity 91.3%, specificity 72.7% [16]), IVIM (sensitivity 89.0%, specificity 93.4%) [21], and nuclear medicine studies including 11C MET-PET (sensitivity 82.0%, specificity 75.0%) [24]. Other nuclear medicine studies including 18F-FET PET appear to have the highest accuracy with a sensitivity of 95% and a specificity of 91% [23], increasing to 100% and 100%, respectively, for glioma studies [37, 38]. Notably, not all cases within these studies had gold standard histopathological verification of radiation necrosis or tumor progression which may explain some discrepancies to our study [15, 16, 21, 23, 24, 38]. The substantial inter-reader agreement (kappa = 0.61), lack of required region of interest (ROI) delineation, quantification, or incorporation of prior imaging studies for longitudinal assessment suggests the centrally restricted diffusion sign to be a useful tool for the evaluation of radiation necrosis in clinical practice.

Our single timepoint location-based interpretation represents a paradigm shift in the application of DWI for post-radiation brain metastases. Restricted diffusion (high DWI, low ADC) has often been used as a biomarker to represent hypercellularity and thus tumor, while increased or increasing diffusivity (low DWI, high ADC) was considered to represent necrosis, pseudoprogression, edema, or treatment response [14, 25, 28, 29]. Huang et al. presented a longitudinal imaging study on brain metastases treated with SRS through MRI studies performed at one week, one month, and three months post therapy to predict treatment success [29]. Higher mean ADC values were reported in the group demonstrating radiation-induced central necrosis based on ROIs encompassing entire lesions. A longitudinal study by Chen et al. focusing on changes in ADC values described decreased or unchanging ADC indicative of non-response [28]. Knitter et al. evaluated the performance of DWI to differentiate true progression from pseudoprogression using pre- and post-treatment MRIs [14]. Here, ADC values between the two groups were not significantly different at any single timepoint, but an interval increase in ADC value was reported to identify pseudoprogression in 77% of lesions [14]. In the latter two studies [14, 28], DWI/ADC was assessed within the contrast enhancing regions only, with areas of necrosis excluded from the ROIs as they were not considered to contain relevant information, a methodology also seen in previous glioma studies [39, 40].

Shifting from a quantitative assessment to a qualitative, visual interpretation of DWI, Cha et al. differentiated radiation necrosis from tumor progression in SRS treated brain metastases using differing imaging patterns [20]. In their study, radiation necrosis was found only in their “3 layer” pattern, with diffusion restriction located in the middle layer, corresponding to coagulative necrosis histologically. This methodology is similar to our study in that radiation necrosis was assessed for using visual analysis of imaging patterns alone without quantification or lesion segmentation. One main difference is that we did not differentiate layers within the contrast-enhancing rim, in line with previous glioma studies assessing for radiation necrosis using the centrally restricted diffusion sign [30, 31]. The association between diffusion restriction and radiation necrosis has been shown in prior studies [20, 30] and rat models [41] and appears to be due to coagulative necrosis with fibroblastic proliferation, hemosiderin laden macrophages, and inflammatory cells [34]. Comparisons have been drawn to a combination of a non-infectious pus-like material with abundant biological borders restricting diffusivity on a molecular scale [20].

For tumor progression, opposing findings have been described in the literature with regards to central diffusivity. While some previous studies associate absent diffusion restriction within the necrosis with tumor progression [30, 31, 41], Cha et al. found all 5 cases of centrally restricted diffusion to represent tumor progression in their 16 subject cohort. Clearly, there is a discrepancy with regards to the diffusivity of the central necrosis in tumor progression cases which was also seen in our study, with central restriction seen in 13/36 (35%) tumor progression cases and absent central restriction seen in the remaining 23/36 (65%) tumor progression cases. Based on Cha et al.’s published data, we were not able to explain their discordant findings compared with our study [20]. On histopathological sub-analysis of our tumor progression cases, no differences were found between centrally restricting and centrally non-restricting cases with regards to histopathologic tumor fraction, patient age, time from SRS to MRI, or radiation dose. The true correlation between varying DWI signal characteristics in tumor progression is likely multifactorial and due in part to a combination of tumor characteristics, cell membrane density, macromolecular size and type, tissue oxygenation and viscosity, cell radiosensitivity, perfusion, and stage of evolution of necrosis [39, 42].

In this study, we only included histopathologically proven cases, which led to the small patient cohort. Histopathology did not differentiate lesion subsite based on MRI. Second, a large proportion of potential patients were excluded as they did not have ring-enhancing metastases or had increased susceptibility within the lesion, limiting the usefulness of the sign to a subset of patients. Finally, our cohort is heterogeneous with regards to treatment regimen and MR imaging technique, factors which are due to the retrospective nature of the study, however we feel this to be representative of what is seen on a daily basis.

To conclude, we found a low probability of radiation necrosis in the absence of the centrally restricted diffusion sign for brain metastases treated with SRS. The high negative predictive value of the centrally restricted diffusion sign combined with its substantial inter-reader agreement, lack of required region of interest delineation, quantification, or incorporation of prior imaging studies for longitudinal assessment suggests this to be a useful tool in clinical practice and adds to the growing body of evidence for differentiation of radiation necrosis from tumor progression. In future research, a multiparametric model incorporating the centrally restricted diffusion sign with advanced MR imaging techniques such as DSC Perfusion and IVIM may provide higher accuracies for the discrimination of radiation necrosis from tumor progression.

## Data Availability

Study data is available upon reasonable request.

## Abbreviations

ADC: Apparent diffusion coefficient
DCE: Dynamic contrast enhanced
DSC: Dynamic susceptibility contrast
DWI: Diffusion weighted imaging
IR-FSPGR: Volumetric T1-weighted fast spoiled grass sequence with IR preparation
MP-RAGE: T1-weighted magnetization prepared rapid gradient echo
NPV: Negative predictive value
PACS: Picture archiving and communication system
PPV: Positive predictive value
ROI: Region of interest
STARD: Standards for the Reporting of Diagnostic Accuracy Studies
SRS: Stereotactic radiosurgery
SWI: Susceptibility weighted image
WBRT: Whole brain radiotherapy
